# O.5.2-10 Barriers to physical activity among parous women postpartum: a qualitative study in Denmark

**DOI:** 10.1093/eurpub/ckad133.251

**Published:** 2023-09-11

**Authors:** Solvej Videbæk Bueno, Dagmar Clausen, Vassilis Sevdalis, Rasmus Østergaard Nielsen

**Affiliations:** Research Unit for General Practice, Aarhus, Denmark; Department of Public Health, Aarhus University, Aarhus, Denmark; Department of Food and Nutrition and Sports Science, University of Gothenburg, Gothenburg, Sweden; solvej.videbaek@ph.au.dk; Research Unit for General Practice, Aarhus, Denmark; Department of Public Health, Aarhus University, Aarhus, Denmark

## Abstract

**Purpose:**

Physical activity (PA) among parous women postpartum has multiple health benefits to mother and child. Knowledge of the barriers to PA among parous women postpartum is necessary to support future health-enhancing PA interventions. The purpose of this study was to explore the barriers to PA in parous women six to 10 months after giving birth.

**Methods:**

In this qualitative study, we used a phenomenological–hermeneutic approach to explore barriers to PA among parous women six to 10 months postpartum. Semi-structured interviews were conducted including 16 parous women from Central Denmark Region. These women were physically active before pregnancy; had a mean age of 29 years; differed in level of education; and represented both vaginal birth and caesarian section. The women were recruited from two sources. 1) a video on Facebook, which had >10.000 views, and 2) the Department of Health Visitors at Aarhus Municipality. Nvivo was used for analysis. We used open and closed coding, and quotes from the interviews were used to support claims and illustrate the identified barriers.

**Results:**

Three categories of barriers were identified. 1) The individual level: lack of motivation to engage in PA, insufficient self-efficacy in PA, limited economical resources, and bodily physical changes. 2) The interpersonal level: lack of a training partner, childcare, and missing community-based activities in sports clubs. 3) The structural level: lack of guidance and directions on PA from the healthcare system, lack of training facilities, the accessibility of training facilities, and the weather. Fourteen of the 16 included women felt “lack of guidance on PA from the healthcare system” as a barrier to PA postpartum.

**Conclusions:**

11 barriers to PA among parous women were identified. On a policy level, “lack of guidance and directions from the health care system” is a modifiable barrier, calling for intervention. Health-enhancing PA in parous women postpartum may be improved by addressing this barrier, when future maternity care programs are developed.

**Support/Funding:**

No funding source was applied for this study.

